# Is Dietary 2-Oxoglutaric Acid Effective in Accelerating Bone Growth and Development in Experimentally-Induced Intrauterine Growth Retarded Gilts?

**DOI:** 10.3390/ani10040728

**Published:** 2020-04-22

**Authors:** Ewa Tomaszewska, Piotr Dobrowolski, Małgorzata Świątkiewicz, Janine Donaldson, Iwona Puzio, Siemowit Muszyński

**Affiliations:** 1 Department of Animal Physiology, Faculty of Veterinary Medicine, University of Life Sciences in Lublin, Akademicka St. 12, 20-950 Lublin, Poland; iwona.puzio@up.lublin.pl; 2 Department of Functional Anatomy and Cytobiology, Faculty of Biology and Biotechnology, Maria Curie-Sklodowska University, Akademicka St. 19, 20-033 Lublin, Poland; piotr.dobrowolski@umcs.lublin.pl; 3 Department of Animal Nutrition and Feed Science, National Research Institute of Animal Production, Krakowska St. 1, 32-083 Balice, Poland; malgorzata.swiatkiewicz@izoo.krakow.pl; 4 School of Physiology, Faculty of Health Sciences, University of the Witwatersrand, 7 York Road, Parktown, Johannesburg 2193, South Africa; janine.donaldson@wits.ac.za; 5 Department of Biophysics, Faculty of Environmental Biology, University of Life Sciences in Lublin, Akademicka St. 13, 20-950 Lublin, Poland

**Keywords:** intrauterine growth retardation, 2-oxoglutaric acid, pig, bone development, articular cartilage, immunohistochemistry

## Abstract

**Simple Summary:**

Intrauterine growth restriction (IUGR) is a significant health issue that not only affects infant mortality or term body weight, but may also predispose individuals to a reduced rate of weight gain and the development of numerous diseases later in life. In livestock production, growth restricted (IUGR) animals require more time to reach slaughter weight. In this study, we examined the effects of long-term administration of 2-oxoglutaric acid (2-Ox) to experimentally-induced intrauterine growth retarded gilts.

**Abstract:**

In this study, the effect of long-term 2-oxoglutaric acid (2-Ox) supplementation to experimentally-induced intrauterine growth retarded gilts was examined. Sows were treated with synthetic glucocorticoid (dexamethasone) every second day, during the last 45 days of pregnancy, at a dose of 0.03 mg/kg b.w. At birth, the gilts were randomly divided into two groups: unsupplemented and supplemented with 2-Ox for nine months (0.4 g/kg body weight/day). Oral supplementation of 2-Ox to experimentally-induced intrauterine growth retarded gilts increased body weight at weaning as well as final body weight at the age of nine months, and showed a regenerative effect on bone mineralization and morphology of trabeculae and articular cartilage. The positive effects on bone structure were attributed to the 2-Ox-induced alterations in bone metabolism, as evidenced by the changes in the expression of proteins involved in bone formation and remodeling: osteocalcin (OC), osteoprotegerin (OPG), receptor activator of nuclear factor kappa-Β ligand (RANKL), tissue inhibitor of metalloproteinases 2 (TIMP-2), bone morphogenetic protein 2 (BMP-2), cartilage oligomeric matrix protein (COMP), and vascular endothelial growth factor (VEGF).

## 1. Introduction

Fetal growth and development involves complex biological processes dependent on a variety of genetic and epigenetic factors. The effects of these epigenetic factors on cellular and physiological phenotypic traits may result from external or environmental factors (stress of being pregnant, infections), or they may be part of normal development. Epigenetic factors have been shown to affect placental size and function, placental blood flow, nutrient transfer and supply of oxygen from the mother to the fetus, availability of nutrients to the fetus, and fetal hormonal balance. Changes in fetal nutrition and endocrine status can in turn affect adaptive developmental, structural, physiological, and metabolic functions, as well as inhibit postnatal growth. Moreover, the above mentioned changes can lead to intrauterine growth retardation (IUGR), which results in piglets with significantly lower body weights [1,2,3,4,5]. Moreover, IUGR is closely associated with a reduction in gastrointestinal tract mass, altered patterns of enzyme maturation, decreased secretion of insulin, lowered proliferation and extensive apoptosis of pancreatic β-cells, hepatic histomorphometric changes, elevated blood pressure, endocrine or metabolic disorders, obesity, behavioural changes, and changes in bone metabolism later in life [6,7,8,9,10,11]. In addition, IUGR is associated with a phenomenon called catch-up growth, where a greater growth rate than that expected for a specific age is observed, following the removal of the growth-inhibiting conditions. Growth-retarded newborns can reach the same size (body weight) during postnatal development as piglets that were not growth retarded [12].

Glucocorticoids (GCs) play an important role in prenatal development, specifically with regards to prenatal hormonal regulation and ensuring the proper development of the nervous system. In some animal species such as rats, rabbits, and mice, these processes mainly occur during the postnatal period. In sheep, guinea pigs, and primates, the development and functional maturation of the nervous and endocrine systems occurs during the last few weeks of fetal life [13]. Glucocorticoids are also important hormonal factors in the development of IUGR. High concentrations of endogenous GCs are observed during the fetal period, following either short-term or chronic stress of a pregnant female (transport stress, social stress, and abnormal living conditions). Studies on prenatal programming have shown that GCs primarily inhibit fetal growth and reduce placental size, depending on the concentration of GCs and the duration of exposure [14,15]. There are several animal models of experimentally-induced IUGR, one of which involves the use of GCs. Some studies have shown that administration of GCs prenatally transiently or permanently inhibited longitudinal bone growth in piglets [16,17]. Previous studies on pigs have also shown that maternal GCs administration alters the quality of articular cartilage of offspring in a gender-dependent manner [17,18,19,20].

Connective tissue homeostasis, which is regulated by many factors, varies in prenatal and postnatal life and can be affected by several hormones and nutritional factors [21,22,23,24,25]. The quality of food consumed by pregnant dams, or by their offspring during postnatal life, plays an important role in fetal growth and the subsequent development of newborns. Specific nutritional ingredients can play particular roles as functional foods in animal husbandry. It is well known that nutrition plays an important role in the management of many diseases and in the structural development of mammals. Nutrition during early life can also program for long-term effects in later life. The precursor of glutamine, proline, and hydroxyproline, 2-oxoglutaric acid (2-Ox), has been shown to have a protective effect against GC-induced prenatal programming of the intestine or bone development, including increases bone mass and the strength [11,19,26,27,28,29]. This nutritive protective role of 2-Ox is known from in vitro, human, and animal model experiments [23,30,31,32,33,34,35].

Studies evaluating the long-term effects of maternal GCs overload, as well as the effects of long-lasting treatment with 2-Ox on bone cartilage in gilts, are limited. Earlier studies have shown that newborn and neonatal male growth-retarded pigs are more affected than females [18,20,36]. Therefore, an experiment was designed to evaluate if 2-Ox given during postnatal life accelerates the growth and bone development in GC-induced intrauterine growth-retarded gilts.

The aim of this study was to investigate the effects of long-term, postnatal dietary administration of 2-Ox on the local structure of the growth plate and articular cartilage, the chondrocyte activity in the growth plate cartilage, and osteocyte activity in trabeculae, as well as the quantity and quality of cancellous bone in experimentally-induced (by maternal GCs overload), intrauterine growth-retarded, mature gilts. For this purpose, the present study involved the combined use of multiple methods, including mechanical testing, dual X-ray absorptiometry (DXA), and light microscopy, in combination with histochemical and immunohistochemical methods to characterize the protective role of 2-Ox in IUGR.

## 2. Materials and Methods

### 2.1. Ethical Approval

All experimental procedures were approved by The Local Ethics Committee on Animal Experimentation of the University of Life Sciences in Lublin, Poland (40/2005-2007) and complied with the Directive 2007/526/EC of the European Parliament and of the Council on the protection of animals used for scientific purposes. Throughout the experimental period, the health status of pregnant sows and piglets was regularly monitored by a veterinarian. Besides suckling behavior, pig behavior was not monitored.

### 2.2. Pregnant and Lactating Sows

Six clinically healthy, multiparous (second parity) sows of the Large White Polish breed were sired by the same boar and singly housed in separate cages, from the 70th day of pregnancy. Two weeks before parturition, the sows were moved into a farrowing room and kept in individual pens. During the experimental period, pigs were kept under standard rearing conditions (controlled temperature and humidity), with free access to fresh water. Sows were fed twice a day with a properly balanced feed mixture (Table 1). Sows were fed an amount of 3.0 kg of feed mixture per sow during late gestation. During lactation, the feeding rate was dependent on the litter size. The feed mixture was formulated to meet or exceed the requirements for sows with regards to nutrients, metabolizable energy, and mineral elements, during pregnancy and lactation [37]. The feed mixture used for sows during pregnancy and lactation contained the following in 1 kg: 13.2 MJ ME, 162 g of crude protein, and 9.5 g of lysine, and the scheme of the main essential amino acids proportion was lysine 100%/methionine + cysteine 60%/threonine 65%/tryptophan 22%.

**Figure 1:**
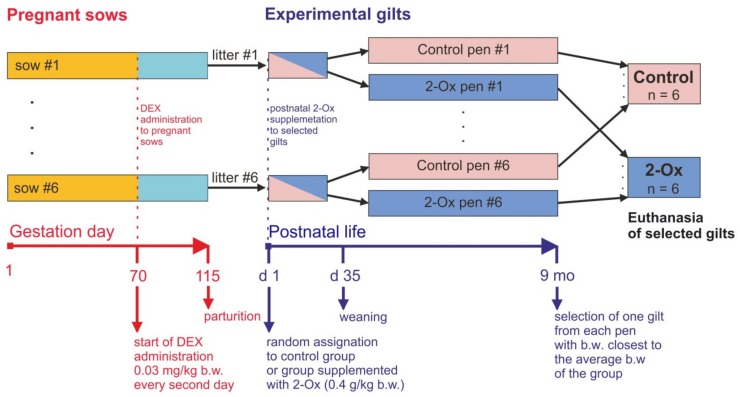
Scheme showing the experimental design. From the 70th day of gestation to parturition, dexamethasone (DEX) was administered to each sow at the dose of 0.03 mg/kg body weight (b.w.) every second day. After delivery, all newborn gilts were randomly assigned to one of the two experimental groups: the control group (not administered with **2-oxoglutaric acid** (2-Ox)) or the group supplemented with 2-Ox with the dose 0.4 g/kg b.w. daily for nine months (2-Ox group). After weaning, the gilts were kept in group pens, with each litter in a separate pen. After final weighing at the age of nine months, one gilt from each litter/pen with a body weight closest to the average body weight of the experimental group was selected and euthanized.

From the 70th day of pregnancy, a synthetic glucocorticoid (dexamethasone, DEX) was administered to all sows to experimentally induce symptoms of intrauterine growth retardation (IUGR) in their offspring (Figure 1). Dexamethasone (Eurovet Animal Health B.V., Bladel, Netherlands) was administered to the sows via intramuscular injection in the morning (0.03 mg/kg body weight/every second day). The total DEX dose was about 75 mg per pregnant GC-treated sow, during the last 45 days of pregnancy. The dose and period of DEX treatment were determined from previous studies [12,17,19]. These studies have shown that DEX administration did not influence gestation length or the mean number of stillborn and live born piglets in litters delivered from GC-treated sows when compared with the sows not treated with GCs [12,17,19]. Therefore, in the present study, the group of sows not treated with GCs was excluded, in accordance with the “3Rs” principle and the Ethics Committee recommendation, in order to avoid unnecessary use of experimental animals.

### 2.3. Offspring

All piglets (67 in total, including 31 gilts and 38 barrows) were born at term via natural birth. The number of live-born offspring per litter from individual sows did not differ statistically (no pigs were stillborn). At birth, unsuckled gilts were weighed, marked, and examined by a veterinarian. Owing to the absence of weight outliers (determined using the means of Grubbs’ test statistic) in each litter, all gilts were randomly assigned to one of the two experimental groups: the control group (not administered with 2-Ox) or the group orally supplemented with 2-Ox (2-Ox group) (Figure 1). The control group consisted of a total of *n* = 16 gilts, while the number of gilts assigned to the 2-Ox group was *n* = 15. Until weaning at the age of 35 days, all piglets were housed with their own mothers and not translocated between sows. After weaning, the gilts were kept in group pens, with each litter in a separate pen. The piglets were kept under standard rearing conditions with free access to fresh water, and immune procedures were conducted in accordance with the standard requirements of the pig farm. Offspring were fed standard feed mixtures in accordance with their production stage (Table 2). All diets were formulated to meet or exceed the requirements for piglets with regards to for nutrients, metabolizable energy, and mineral elements [37]. The feed mixture used for piglets during the nursing and weaning periods contained the following in 1 kg: 13.8 MJ ME, 185 g of crude protein, and 12.8 g of lysine, and the scheme of the main essential amino acids proportion was lysine 100%/methionine + cysteine 60%/threonine 64%/tryptophan 20%.

All gilts were weighed every week. The gilts from both groups were kept to the age of nine months. After final weighing, *n* = 6 gilts from each experimental group were selected (i.e., one gilt from each litter/pen with a body weight closest to the average body weight of the group) and fasted for 24 h, after which blood samples were collected. Blood was collected from the subclavian vein using a standard venipuncture into 6 mL Vacutest tubes without anticoagulant. The blood samples were allowed to stand at room temperature to allow the blood to clot, after which the samples were centrifuged at 3000× *g* for 15 min. The serum was collected and stored in Eppendorf tubes at −25 °C until assayed. After blood collection, the piglets were euthanized by an intravenous injection of a lethal dose of *Pentobarbitalum natricum* (Morbital, Biowet, Puławy, Poland).

During the neonatal period, gilts from the 2-Ox group received 2-Ox per os each morning, while gilts from the control group received PhS per os at the same volume as the gilts receiving 2-Ox. After weaning, the solutions were administered together with the diet, starting from the 36th day of life to the end of the experimental period. The 2-Ox group received 2-Ox at a dose of 0.4 g/kg body weight in 2 mL of the solution prepared from powdered 2-Ox (2-Ox; Protista International AB, Sweden) of 99% purity, which was mixed with distilled water to obtain a solution. The pH of the stock solution was buffered by the addition of NaOH to obtain a final pH of 7.3. The dosage of 2-Ox used in this study was the same as that previously used in studies involving pigs [8,9,10,11,12,19,20,29,30,31,32,33,34,38].

### 2.4. Serum Biochemical and Hormonal Analysis

Serum lipid profile (total cholesterol, low-density lipoproteins, high-density lipoproteins, and triacylglycerols), urea, creatinine, uric acid, albumin, alanine transaminase (ALT), aspartate transaminase (AST), alkaline phosphatase, lactate dehydrogenase (LDH), and selected minerals (Fe, Ca, P, and Mg) were determined using an automatic biochemistry analyzer (Mindray BS-120, Bio-Medical Electronics, Shenzhen, China). Test kits were purchased from BioMaxima (Lublin, Poland) and all analysis procedures were verified with the use of multiparametric control serum (BioCal, BioMaxima, Lublin, Poland).

Serum concentrations of insulin-like growth factor 1 (IGF-1) were determined using an enzyme-linked immunosorbent assay kit (ELISA; Uscn Life Science Inc., Wuhan, China) with a minimum detectable concentration of 7.8 ng/mL, and a Benchmark Plus microplate spectrophotometer (Bio-Rad Laboratories, Inc., Hercules, CA, USA). All samples were analysed in duplicate.

Determination of free amino acid concentrations in serum was performed with the use of ion-exchange chromatography and an INGOS AAA-400 apparatus for the automatic analysis of amino acids (INGOS Corp., Prague, Czech Republic).

### 2.5. Bone Analysis

Immediately after euthanasia, the femora from individual pigs were dissected, cleaned from adherent tissues, wrapped in gauze soaked in PhS, and frozen at −20 °C until further analyses. In the subsequent stages of analyses, the right femora were earmarked for bone midshaft geometry measurements and histomorphometric analysis, while the left femora were used for determination of bone weight, length, densitometry, and mechanical testing. Before all analyses, the frozen bones were thawed overnight in the laboratory at 10 °C.

Right femora were cut across in the midpoint of the bone diaphysis with a diamond bandsaw (MBS 240/E, Proxxon GmbH, Foehren, Germany). Bone diaphysis geometric properties, such as bone diaphysis cross-sectional area, mean relative wall thickness, cortical index, and cross-sectional moment of inertia, were determined on the basis of measurements of bone cross-sectional diameters (internal and external) measured using a digital caliper [39].

The measurement of bone mineral density (BMD) and bone mineral content (BMC) for the whole bone was performed using the dual-energy X-ray absorptiometry (DXA) method on a Discovery W densitometer (Hologic, Bedford, MA, USA), which was calibrated prior to the measurements using bone phantoms of known BMD [22].

The mechanical properties of bones were determined using the three-point bending test performed on a Zwick Z010 universal testing machine (Zwick-Roell GmbH & Co., Ulm, Germany). The bones were loaded in the midpoint of the bone diaphysis in the cranial-caudal plane with a constant loading rate of 10 mm/min until bone fracture. The ultimate strength was registered and the ultimate stress (bone material trait) was calculated using the appropriate engineering beam-theory equation [40].

### 2.6. Histomorphometry

Cylindrical 20 mm thick samples of the lateral condyle were cut using a diamond bandsaw (MBS 240/E, Proxxon GmbH, Foehren, Germany), perpendicularly to the articular surface, from the same anatomical position from the middle of the of distal epiphysis. Samples were fixed in phosphate-buffered 4% paraformaldehyde for 24 h at room temperature, decalcified in EDTA solution (10%, pH 7.4), dehydrated through a graded ethanol series, and embedded in paraffin. Sections were cut with a microtome at a thickness of 4 μm, mounted on SuperFrost Plus slides (Thermo Scientific, Schwerte, Germany), and processed for routine staining procedures and immunohistochemistry. In total, nine slides from each piglet were prepared. Slides were stained with Safranine O, Goldner’s trichrome, and Picrosirus red (PSR) stains in order to evaluate proteoglycan content in the articular cartilage, basal morphology of the articular and growth plate cartilage, and the distribution of immature (thin) and mature (thick) collagen fibres, respectively [41,42,43]. Stained slides were observed in normal (Safranine O, Goldner’s trichrome) and polarized (PSR) light using a light microscope (CX43, Olympus, Tokyo, Japan).

The analysis of the images collected was performed using CellSens software (Olympus, Tokyo, Japan). For the articular and growth plate cartilage, total thickness and the thicknesses of their main zones were measured. Individual layers of articular cartilage (superficial, transitional, and radial) were identified as previously described by Pearle et al. [44]. For the growth plate cartilage, four zones (reserve, proliferative, hypertrophic, and ossification) were identified [45,46]. All measurements were performed at eight sites along the articular and growth plate cartilage and an average was reported [44,45,46].

To assess the morphology of trabecular bone, the relative bone volume (BV/TV), trabecular thickness (Tb.Th), trabecular separation (Tb.Sp), and trabecular number (Tb.N) were calculated for the microscopic images of the bone epiphysis and metaphysis using ImageJ software [29].

The percentage of immature collagen as a proportion of total collagen content in PSR stained sections was calculated using the pixel counting method, using a color threshold tool in ImageJ software (version 1.50b, National Institutes of Health, Bethesda, MD, USA) [47] to calculate the area of red (mature) and green (immature) collagen fibres in selected image sections.

### 2.7. Immunohistochemistry

Immunohistochemical staining of decalcified sections was performed according to a previously described protocol [21]. Briefly, slides were dewaxed and hydrated before blocking cellular peroxidase activity with 3% H_2_O_2_ (Sigma-Aldrich, St. Louis, MO, USA). Antigen retrieval step was performed by 10 min enzymatic retrieval with proteinase K (Sigma-Aldrich, St. Louis, MO, USA) at 37 °C.

Swine-specific primary antibodies targeting osteocalcin—OC (Abcam, Cambridge, UK, dilution 1:100), glucocorticoid receptor—GR (Santa Cruz Biotechnology, Santa Cruz, CA, USA, dilution 1:50) osteoprotegerin—OPG (Abcam, Cambridge, UK, dilution 1:100), receptor activator of nuclear factor kappa-Β ligand—RANKL (Biorbyt, USA, dilution 1:50), tissue inhibitor of metalloproteinases 2—TIMP-2 (Abcam, Cambridge, UK, dilution 1:100), bone morphogenetic protein 2—BMP-2 (Abcam, Cambridge, UK, dilution 1:250), vascular endothelial growth factor—VEGF (Biorbyt Ltd., Cambridge, UK, dilution 1:50), and cartilage oligomeric matrix protein—COMP (Elabscience, USA, dilution 1:100) were incubated overnight at 4 °C in blocking buffer (TBS with 5% goat serum; Sigma-Aldrich, St. Louis, MO, USA). The next day, peroxidase conjugated goat anti-rabbit (Rockland Immunochemicals, Inc. Pottstown, PA, USA, dilution 1:500) secondary antibody was incubated for 30 min. The sections were developed in the DAB substrate (3, 3′ diaminobenzidine tetrahydrochloride, Abcam, Cambridge, UK), which is a substrate-staining chromogen. Slides were counterstained with haematoxylin (Sigma-Aldrich, St. Louis, MO, USA) and mounted with DPX mountant (Sigma-Aldrich, St. Louis, MO, USA). Negative control sections for each antibody were obtained by identical immunohistochemical staining, excluding the addition of the primary antibody. Slides were examined using a CX43 microscope (Olympus, Tokyo, Japan).

### 2.8. Statistical Analysis

All results are expressed as means ± SD (standard deviation). On graphs, raw data are also presented as individual data points. The statistical analyses of the data were performed using Statistica 13 software (Dell Software Inc., Austin, TX, USA). The normality of data distribution was tested using the Shapiro–Wilk test and equality of variance was tested by the Levene’s test. A comparison between normally distributed variables was carried out using a two-tailed Student’s *t*-test. If there was a lack of normal distribution and/or unequal variance of data, the Mann–Whitney *U* test was applied. For all tests, *p* < 0.05 was considered statistically significant.

## 3. Results

### 3.1. Body Weight

**Figure 2:**
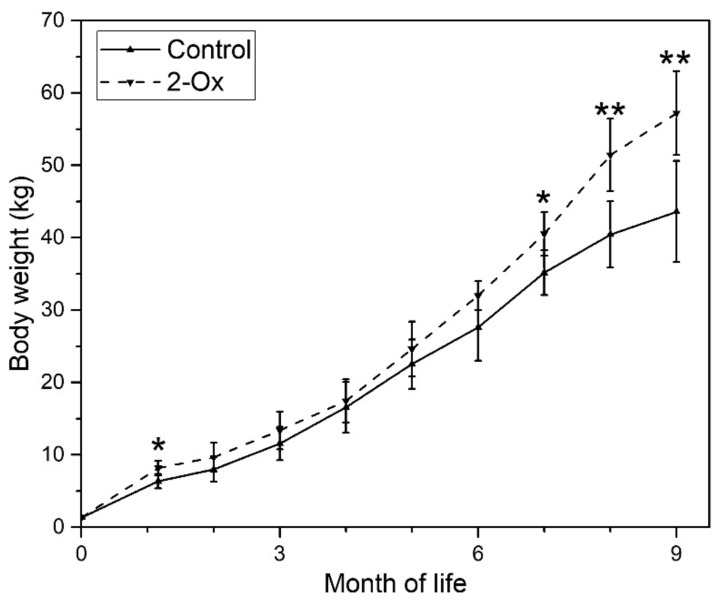
The effect of dietary supplementation of 2-Ox on body weight in glucocorticoid (GC)-induced retarded mature gilts. Control—the control group included retarded gilts not supplemented with 2-Ox; 2-Ox—the 2-Ox group included retarded gilts supplemented with 2-Ox. Data are mean values ± SD. Statistical significance: * *p* < 0.05; ** *p* < 0.01 (two-tailed Student’s *t*-test).

Mean term body weight was 1328 ± 103 g. At the weaning at the age of 35 days, gilts in the 2-Ox group were heavier than controls (6.33 ± 1.32 kg and 8.16 ± 1.02 in the control and the 2-Ox group, respectively; *p* < 0.05). In later life, from the age of two months up to the age of six months, the weight of control gilts did not differ significantly from the weight of gilts supplemented with 2-Ox. Next, the body weight increased in 2-Ox supplemented gilts. The final body weight at the age of nine months was significantly lower in the control group than in the 2-Ox group (43.6 ± 6.7 kg and 57.2 ± 5.8 kg in the control and the 2-Ox group, respectively; *p* < 0.01) (Figure 2).

### 3.2. Bone Morphology, Geometry, Density, and Mechanical Properties

**Figure 3:**
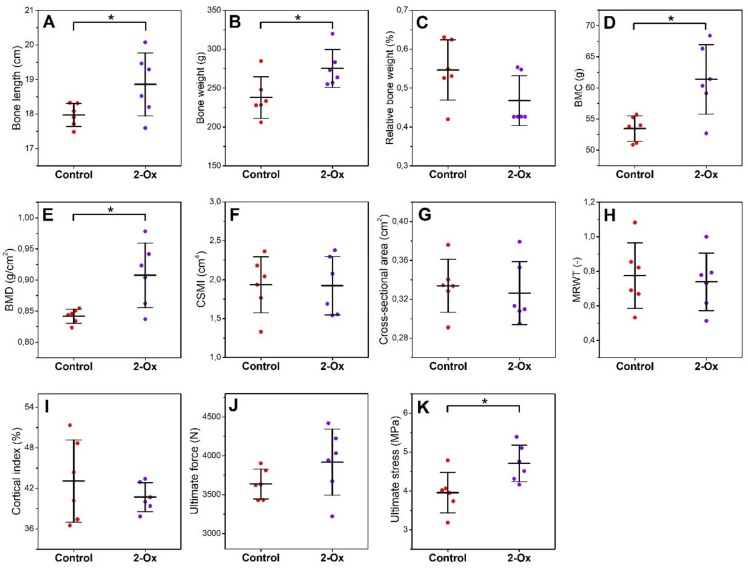
The effect of dietary supplementation with 2-Ox on the geometric and mechanical parameters of femora from glucocorticoid-induced, growth-retarded, mature gilts: (**A**) bone length; (**B**) bone weight; (**C**) relative bone weight; (**D**) bone mineral content (BMC); (**E**) bone mineral density (BMD); (**F**) bone diaphysis cross-sectional moment of inertia (CSMI); (**G**) bone diaphysis cross-sectional area; (**H**) bone diaphysis mean relative wall thickness (MRWT); (**I**) bone diaphysis cortical index; (**J**) ultimate force; and (**K**) ultimate stress. Control—the control group included growth-retarded gilts not supplemented with 2-Ox; 2-Ox—the 2-Ox group included growth-retarded gilts supplemented with 2-Ox at a dose of 0.4 g/kg b.w./day. Data are mean values ± SD (whiskers) from *n* = 6 gilts. Statistical significance: * *p* < 0.05; ** *p* < 0.01 (two-tailed Student’s *t*-test or Mann–Whitney *U* test).

Femora from the prenatally growth-retarded gilts (the control group) were shorter and lighter compared with those from the growth-retarded gilts supplemented with 2-Ox (the 2-Ox group; Figure 3A,B; *p* < 0.05 for both parameters). On the other hand, the relative bone weight did not differ between groups (Figure 3C). Densitometry analysis showed that 2-Ox supplementation increased BMC and BMD in prenatally growth-retarded gilts (Figure 3D,E; *p* < 0.05 for both parameters). No changes in bone midshaft geometry (Figure 3F–I) and raw ultimate force (Figure 3J) were observed. However, supplementation with 2-Ox resulted in an increase in ultimate stress in the prenatally growth-retarded gilts (Figure 3K; *p* < 0.05).

### 3.3. Articular Cartilage, Growth Plate Cartilage, and Trabecular Bone Morphology

**Figure 4:**
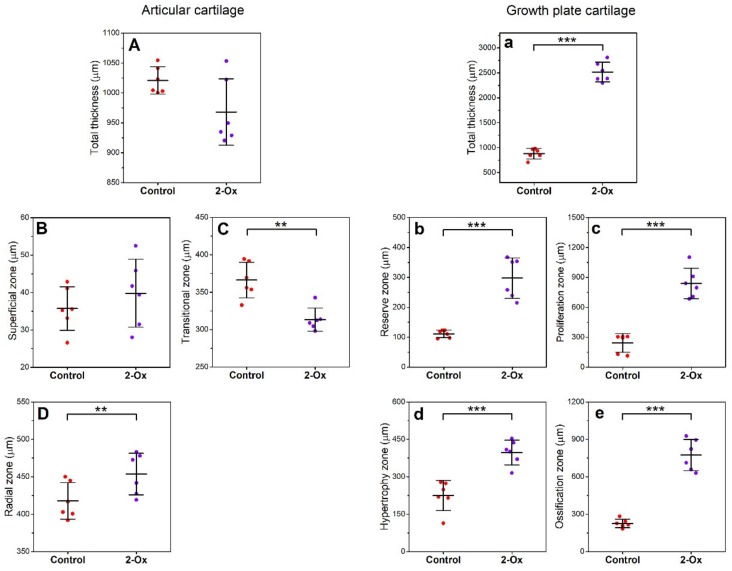
The effect of dietary supplementation with 2-Ox on the thickness of zones in femoral articular cartilage and growth plate cartilage in glucocorticoid-induced, growth-retarded, mature gilts. Articular cartilage: (**A**) total cartilage thickness; (**B**) superficial zone; (**C**) transitional zone; (**D**) radial zone. Growth plate cartilage: (**a**) total cartilage thickness; (**b**) reserve zone; (**c**) proliferation zone; (**d**) hypertrophy zone; (**e**) ossification zone. Control—the control group included growth-retarded gilts not supplemented with 2-Ox; 2-Ox—the 2-Ox group included growth-retarded gilts supplemented with 2-Ox at a dose of 0.4 g/kg b.w./day. Data are mean values ± SD (whiskers) from *n* = 6 gilts. Statistical significance: * *p* < 0.05; ** *p* < 0.01; *** *p* < 0.001 (two-tailed Student’s *t*-test or Mann–Whitney *U* test).

The total thickness of the articular cartilage as well as the thickness of the superficial zone was not different between groups (Figure 4A,B). However, the transitional zone was thinner in the 2-Ox group compared with the control group (Figure 4C; *p* < 0.01) and the radial zone was thicker after 2-Ox supplementation (Figure 4D; *p* < 0.01). The growth plate cartilage was thicker in the 2-Ox group (Figure 4a; *p* < 0.001), with the thickness of each zone reaching higher values following 2-Ox supplementation (Figure 4b–e; *p* < 0.001 for each parameter).

**Figure 5:**
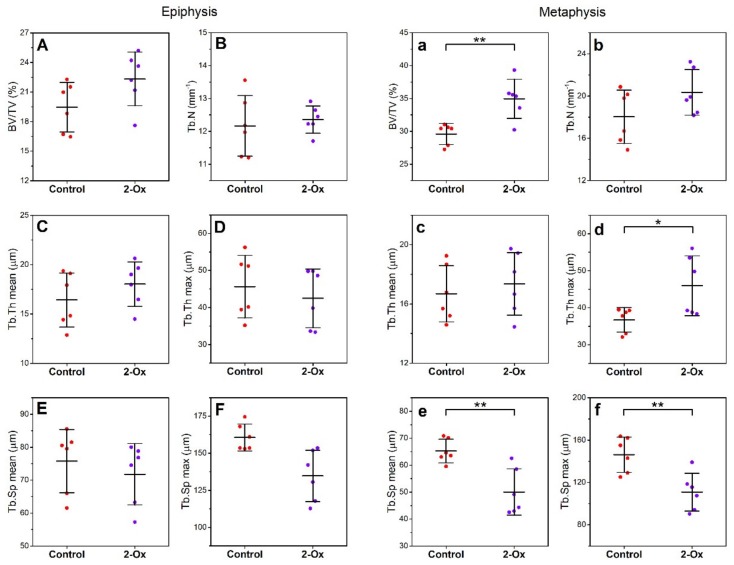
The effect of dietary supplementation with 2-Ox on the histomorphometry of femoral trabeculae in bone epiphysis (**A**–**F**) and metaphysis (**a**–**f**) in glucocorticoid-induced, growth-retarded, mature gilts. BV/TV—relative bone volume; Tb.N—trabecular number; Tb.Th—trabecular thickness; Tb.Sp—trabecular separation. Control—the control group included growth-retarded gilts not supplemented with 2-Ox; 2-Ox—the 2-Ox group included growth-retarded gilts supplemented with 2-Ox at a dose of 0.4 g/kg b.w./day. Data are mean values ± SD (whiskers) from *n* = 6 gilts. Statistical significance: * *p* < 0.05; ** *p* < 0.01 (two-tailed Student’s *t*-test or Mann–Whitney *U* test).

Histomorphometrical parameters of trabecular bone in the epiphysis did not differ between groups (Figure 5A–F). In the metaphysis, 2-Ox treatment in GC-induced growth-retarded gilts resulted in increased relative bone volume (BV/TV; Figure 5a; *p* < 0.01). Although mean trabecular thickness did not change after 2-Ox supplementation (Figure 5c), an increase in maximal trabecular thickness (Figure 5d; *p* < 0.05) and decrease in mean and maximal trabecular space were observed in the metaphysis (Figure 5e,f; *p* < 0.01 for both parameters). Postnatal administration of 2-Ox to gilts prenatally overloaded with GCs did not alter trabecular number (Figure 5b).

**Figure 6:**
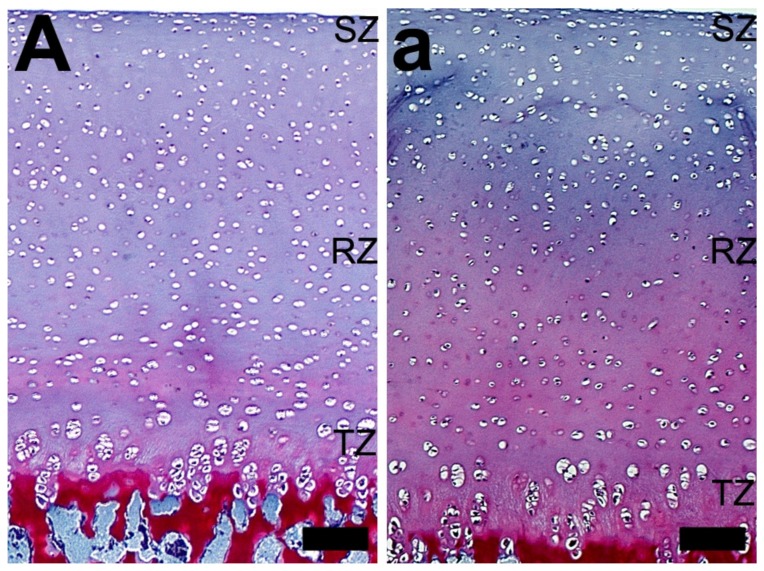
The effect of dietary supplementation with 2-Ox on the femoral articular cartilage structure in glucocorticoid-induced, growth-retarded, mature gilts (Safranine O staining). (**A**) The articular cartilage of the control group (growth-retarded gilts not supplemented with 2-Ox). (**a**) The articular cartilage of the 2-Ox group (growth-retarded gilts supplemented with 2-Ox at a dose of 0.4 g/kg b.w./day). Differences were observed in the distribution and staining intensity of proteoglycans in the femoral articular cartilage (darker Safranine O staining is associated with a higher proteoglycan content). The zones of the articular cartilage: superficial zone (SZ), transitional zone (TZ), and radial zone (RZ). Each scale bar shows 50 µm.

In general, Safranine O staining showed less proteoglycan content in the articular cartilage of growth-retarded gilts from the control group, compared with those from the 2-Ox group (Figure 6). However, while there were no evident differences in the concentration of proteoglycans in the articular cartilage of the control gilts (uniform very weak red or pink staining, Figure 6A), a well-marked gradient in the concentration of proteoglycans in the articular cartilage was observed in the articular cartilage of gilts treated with 2-Ox (Figure 6a). In these groups, Safranine O staining showed the gradual increase in the concentration of proteoglycans from the beginning of the transitional zone to subchondral bone. The most intensive staining with Safranine O was observed around chondrocytes from the radial zone (Figure 6a).

Structural information obtained from the analysis of collagen fibres in PSR stained sections showed both types of fibres with the predominance of mature, thick collagen in the articular cartilage in both groups and no differences in the content of immature, thin collagen. No differences in immature collagen content were observed in trabecular bone of the epiphysis, while a decrease in immature collagen content was noted in the metaphysis of gilts supplemented with 2-Ox (*p* < 0.01; Table 3).

### 3.4. Serum Biochemical and Hormonal Analysis

Long-term postnatal supplementation of dietary 2-Ox to growth-retarded gilts significantly reduced serum concentrations of total cholesterol and low-density lipoprotein (Table 4). No significant differences in serum IGF-1 concentrations or in any of the other biochemical parameters assessed were observed (Table 4).

Analysis of free amino acids indicated that 2-Ox administration to growth-retarded gilts significantly increased serum aspartate (*p* < 0.05) and glutamine (*p* < 0.01) concentrations, whereas serum proline (*p* < 0.001), isoleucine (*p* < 0.01), and leucine (*p* < 0.05) concentrations were reduced (Table 5), compared with controls. The concentrations of other free amino acids were not different between groups.

### 3.5. OC, OPG, RANKL, TIMP-2, VEGF, BMP-2, and GR Expression

**Figure 7:**
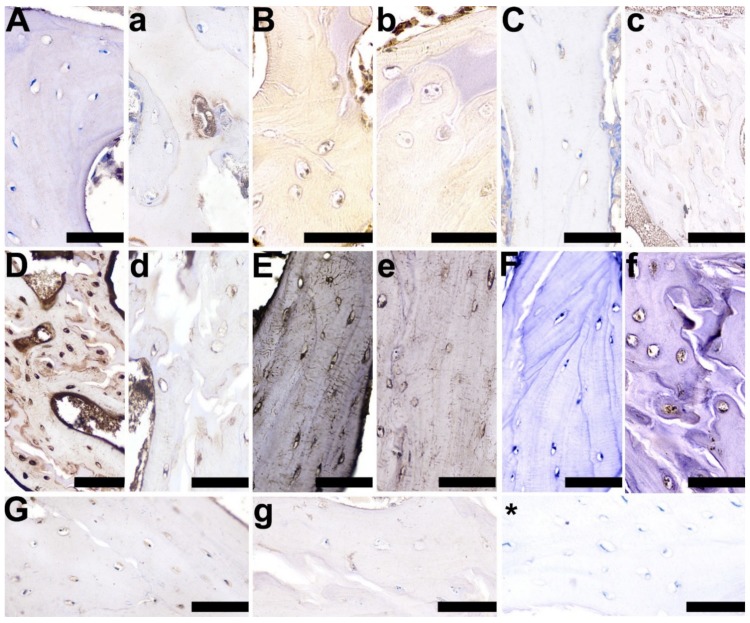
Representative pictures of the immunohistochemical analysis of bone morphogenetic protein 2, BMP-2 (**A**,**a**); glucocorticoid receptors, GRs (**B**,**b**); osteoprotegerin, OPG (**C**,**c**); receptor activator of nuclear factor kappa-Β ligand, RANKL (**D**,**d**); tissue inhibitor of metalloproteinases 2, TIMP-2 (**E**,**e**); osteocalcin, OC (**F**,**f**); and vascular endothelial growth factor, VEGF (**G**,**g**), carried out on formaldehyde-fixed sections from the femoral trabeculae of gilts from the control group (growth-retarded gilts not supplemented with 2-Ox (**A**–**G**)) and the 2-Ox group (growth-retarded gilts supplemented with 2-Ox at a dose of 0.4 g/kg b.w./day (**a**–**g**)). Negative protein expression is represented by blue staining of the cytoplasm in the chondrocytes, while positive protein expression is represented by brown staining in the cells. Additionally, proteins (BMP-2, OC, RANKL, VEGF) secreted and released into the matrix are also stained in a brown color. (*)—anti-body control reaction. Each scale bar shows 50 µm.

Cellular localization of the selected proteins was determined by immunohistochemistry in trabecular bone and the growth plate cartilage. In trabecular bone, the control group displayed an absent or very weak cytoplasmic reaction, and a very weak reaction in the periterritorial zone of osteocytes for OPG (Figure 7C), compared with a well-marked OPG staining in the 2-Ox-treated group (Figure 7c). A very strong positive RANKL expression was observed in the control group (Figure 7D), while a weaker reaction was observed in the 2-Ox group (Figure 7d). Further, the expression of BMP-2, a protein that is involved in the differentiation of osteoblasts from progenitor cells and the induction of bone and cartilage formation during skeletogenesis and regeneration, was not observed in the cells of trabecular bone in the control group (Figure 7A), while a very weak reaction was observed in the cytoplasm and periterritorial zone in single osteocytes of the growth-retarded gilts following 2-Ox treatment (Figure 7a). A strong, positive staining reaction for TIMP-2, a natural inhibitor of matrix metalloproteinases—a group of peptidases involved in the degradation of the extracellular matrix—was observed in the cytoplasm, as well as in the periterritorial zone of all osteocytes in the control group (Figure 7E). A weaker positive, cytoplasmic staining reaction for TIMP-2 was observed in some, but not all cells in the 2-Ox group (Figure 7e). A varying staining reaction for TIMP-2, ranging from absent staining to weak staining and even strong, positive staining, was observed in the periterritorial zone of osteocytes in the 2-Ox group (Figure 7e). A stronger, well-marked reaction for VEGF was noted in the control group, in both the cytoplasm and in the periterritorial zone of the osteocytes (Figure 7G). An absent or weak, cytoplasmic reaction for VEGF was observed in the majority of the cells of the trabeculae from growth-retarded gilts treated with 2-Ox (Figure 7g). No staining reaction for OC was observed in the cytoplasm of the control group (Figure 7F) and a weak, through to moderate, and then to strong cytoplasmic reaction was observed in the 2-Ox group (Figure 7f). Strong staining for GR was observed in the majority of osteocytes in the control group (Figure 7B), while in the 2-Ox group, this reaction was very weak or absent (Figure 7b).

A weak, but well-marked brown BMP-2 staining, representing newly formed bone, was observed in the bone matrix of the 2-Ox group (Figure 7A), compared with a very weak, diffuse staining reaction for BMP-2 in the control group (Figure 7a). A strong, positive RANKL staining reaction was observed in bone matrix of the control group (Figure 7D), with a much lower RANKL expression observed in the matrix of the 2-Ox group (Figure 7d).

**Figure 8:**
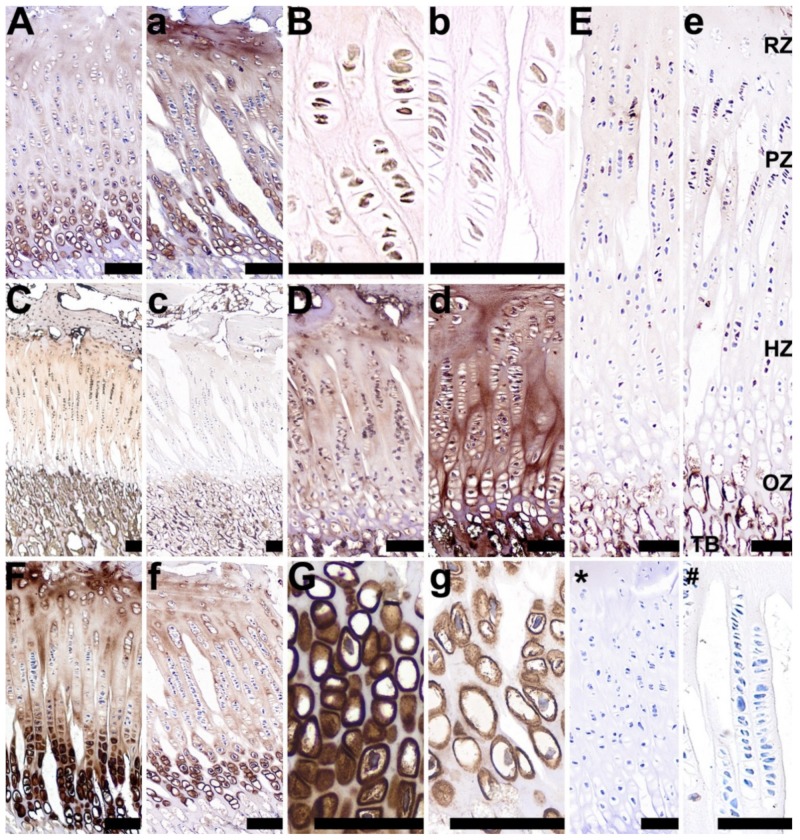
Representative pictures of the immunohistochemical analysis of bone morphogenetic protein 2, BMP-2 (**A**,**a**); glucocorticoid receptors, GRs (**B**,**b**); receptor activator of nuclear factor kappa-Β ligand, RANKL (**C**,**c**); tissue inhibitor of metalloproteinases 2, TIMP-2 (**D**,**d**); osteoprotegerin, OPG (**E**,**e**); and vascular endothelial growth factor, VEGF (**F**,**f**,**G**,**g**), carried out on formaldehyde-fixed sections from the femoral growth plate cartilage in the control group (growth-retarded gilts not supplemented with 2-Ox (**A**–**G**)) and the 2-Ox group (growth-retarded gilts supplemented with 2-Ox at a dose of 0.4 g/kg b.w./day (**a**–**g**)). Negative protein expression is represented by blue staining of the cytoplasm in the chondrocytes, while positive protein expression is represented by brown staining in the cells. Additionally, proteins (BMP-2, OC, RANKL, VEGF) secreted and released into the matrix are also stained in a brown color. (*,#)—anti-body control reaction. In (**e**), the zones of the articular cartilage are labeled as follows: reserve zone (**RZ**), proliferation zone (**PZ**), hypertrophy zone (**HZ**), and ossification zone (**OE**), as well as trabecular bone (**TB**). Each scale bar shows 50 µm.

In the growth plate cartilage, a strong, well-marked brown cytoplasmic signal for RANKL was observed in cells found mainly in the chondrocytes from the resting, proliferative, and hypertrophic zones in the control group (Figure 6C). Moderate RANKL staining was observed in the 2-Ox group, however, this signal was detected in a lower number of cells, found only in the proliferative zone (Figure 8c). VEGF was strongly expressed in the resting and hypertrophic zones in the control group (Figure 8G), whereas the 2-Ox treatment resulted in a weaker staining for VEGF in the same zones (Figure 8g). The proliferative and ossification zones were free from the reaction (Figure 8F,f). Very weak TIMP-2 staining was observed in the cytoplasm of the majority of the chondrocytes and in the growth plate cartilage of the control group (Figure 8D). In turn, TIMP-2 staining was very strong in all chondrocytes of the 2-Ox group (Figure 8d). A moderate signal for OPG was observed in a few cells in the proliferative zone in the control group, with no staining reactions observed in any of the other zones (Figure 8E). 2-Ox administration resulted in a stronger staining reaction for OPG in cells in the proliferative and hypertrophic zones of the growth plate cartilage (Figure 8e). In the control group, moderate BMP-2 expression was noted in the hypertrophic zone, with weak BMP-2 expression in the bone matrix (Figure 8A). The expression of BMP-2 in the matrix of gilts from the 2-Ox group was stronger, especially in the resting zone (Figure 8a). The expression of BMP-2 in the hypertrophic zone in the 2-Ox group was similar to that observed in the control group (Figure 8A,a, respectively). No BMP-2 expression was observed in the ossification zone. Compared with the control group, 2-Ox treatment induced weaker staining for GR in the femoral growth plate cartilage in mature gilts (Figure 8B,b, respectively).

## 4. Discussion

Bone homeostasis is influenced by nutritional and hormonal factors, which vary during the prenatal and postnatal periods. Recent research has focused on the search for natural compounds that are less likely to cause severe negative side effects, while maintaining therapeutic efficiency. The use of compounds such as short-chain peptides or free amino acids is usually preferred as a result of their high rate of absorption from the gastrointestinal tract into the blood circulation, and previous reports have confirmed the absorption of all 2-Ox-derived amino acids from the gastrointestinal tract into the blood, following oral consumption in humans and animals [10,48,49]. Numerous studies have shown a sex-dependent improvement in bone properties following the consumption of 2-Ox, a precursor of the main amino acids found in collagen [11,19,30,32,33,50]. Dietary 2-Ox supplementation has also been shown to improve articular cartilage structure and finishing body weights of growth-retarded boars with IUGR syndrome, by 18.35% compared with those not supplemented with 2-Ox [20]. Still, despite this increase in body weight, adult growth-retarded boars supplemented with 2-Ox weighed about twofold less at the age of nine months compared with control animals and those in the livestock industry. This shows that a catch-up growth of growth-retarded pigs is not always observed.

The term body weight of gilts in our present study was in the normal range. Earlier studies have shown that, in studies in experimentally DEX-induced IUGR, term body weight can be various. Newborns can weigh more or less (1716–1197 g) compared with not retarded controls depending on the DEX dose, period of the pregnancy, and duration of the administration [9,12,15,16,17,18,20,51]. Thus, only reduced rate of weight gain, changes in the skeletal system, or changes in the intestinal tract observed later in life in experimental pigs indicated the existence of IUGR. Phases of growth and development of pigs during postnatal life are well described [30]. Rapid increases in overall body mass, as well as in bone mass mineralization, are observed during the first period of life until weaning. After weaning, body weight begins to stabilize or drop slightly, after which a rapid growth phase is observed [30]. The results obtained showed that 2-Ox supplementation resulted in a slight increase in body weight at the age of 35 days. The period around weaning is usually a phase of rapid growth, during which the growth of piglets can be halted, the occurrence of which is usually associated with reduced feed consumption and temporary malnutrition owing to complex stress factors such as maternal separation, the change from milk to a solid feed mixture, and the change of place and hierarchy in the group. In the present study, the gilts in both groups had similar body weights up to the age of six months, after which accelerated growth was once again observed. The positive actions of 2-Ox were evident in the significantly increased (by 31.2%) final body weight of the growth-retarded group following supplementation, compared with the growth-retarded control group at the age of nine month. Again, as in the case of adult growth-retarded boars from the other study [20], none of the animals in the control or 2-Ox groups reached final body weights characteristic of that for a pig in the livestock industry.

Long-term dietary 2-Ox supplementation resulted in longer and heavier bones in our growth-retarded gilts. The lack of differences in the relative bone weights between the two groups of growth-retarded gilts showed that their growth and development were proportional, irrespective of postnatal supplementation. Moreover, 2-Ox supplementation had no effects on overall metabolism, as most of the other biochemical parameters assessed were within normal physiological range and were not significantly different between groups. Total cholesterol and low-density lipoproteins however, were decreased following long-term dietary supplementation with 2-Ox to growth-retarded gilts. This result is consistent with previous studies performed on newborn or weaned piglets, which demonstrated that 2-Ox can influence lipid metabolism. The lack of influence of dietary 2-Ox on other serum biochemical parameters is also consistent with previous studies [10,29,52], despite the fact that the findings presented in this study relate to mature growth-retarded gilts. On the other hand, our growth-retarded gilts supplemented with 2-Ox demonstrated increased concentrations of asparagine and glutamine, and decreased concentrations of isoleucine, leucine, and proline. This result is partially in agreement with other results, which have also shown an increase in glutamine in growth-retarded piglets [10,18]. Glutamine is an amino acid formed from 2-Ox and belongs to the so-called glutamate family, which includes glutamate and its derivatives proline and arginine. Glutamine has generally been considered to be a non-essential amino acid in health, while in catabolic states and stress, including weaning, it is an essential fuel source for cells of the gastrointestinal tract and rapidly dividing cells in the immune system [10,53]. A positive effect of glutamine supplementation on piglets during the first few days after weaning was observed, including an improvement in growth performance, intestinal morphology, and a strong anti-bacterial activity against *Escherichia coli* and *Clostridium perfringens* [54,55,56]. These beneficial effects of glutamine on digestive tract volume and microbiome, which translate into improved health and digestive efficiency, might partially explain the higher body weight gains noticed in the present study in the 2-Ox pigs. What is more, asparagine produced from glutamate or glutamine is an essential amino acid involved in signaling and neuronal development [57], and can thus determine the general growth and function of living organisms. Further, 2-Ox is an important source of amino acids for collagen synthesis and proper function of muscle tissue [31]. In the current study, a decrease in the concentration of amino acids of the aspartate family, which includes asparagine, methionine, lysine, and isoleucine, was observed. Leucine and isoleucine increase endurance and help heal muscle tissue, preventing the breakdown of muscle proteins caused by injury or stress. Proline is a non-essential amino acid, making up about 17% of collagen, which can be obtained from, for example, glutamate or glutamic acid. It is vital for proper functioning of the cardiovascular system and skin, as well as the musculoskeletal system, including joints and tendons. Moreover, proline is the precursor of hydroxyproline, which contributes to two-thirds of the collagen structure including bone collagen [18]. The decreased lysine and proline concentrations observed in growth-retarded gilts supplemented with 2-Ox could indicate an intensive repair process of connective tissue. Additionally, the increase in thin immature collagen observed in the metaphyseal trabecular bone could also indicate the existence of intensive metabolic processes. Longitudinal bone growth is dependent on growth plate cartilage. Chondrocytes in the resting zone are stimulated to proliferate and then proceed through several maturational stages to hypertrophy, and ultimately build bone. Morphological analysis of the growth plate cartilage in the current study revealed that postnatal supplementation with 2-Ox to growth-retarded gilts resulted in thickening of the growth plate cartilage when compared with the non-supplemented group (Figure 8). Moreover, gilts supplemented with 2-Ox had longer, heavier, and better mineralized femora than the control group.

Postnatal growth is regulated by the somatotrophic axis, which may be affected by GCs during prenatal or postnatal periods, depending on dose, age, and gender. Earlier studies demonstrate that maternal GCs treatment negatively influences the growth plate cartilage in newborn and weaned male piglets, which are growth-retarded [17,19]. 2-Ox administration to male piglets for 35 days during the postnatal period, after maternal GCs overload, improves the thickness of the growth plate cartilage and significantly reduces growth-retardation, suggesting some sort of growth recovery [19].

In the current study, 2-Ox supplementation did not improve any of the indices of bone diaphysis geometry or bone breaking strength in the three-point bending test. However, bone material properties are better traits than raw bone breaking strength in measuring the effect of treatment on bone mechanical strength, as they can correct for bone size and testing procedure [22,25,40]. Higher values of ultimate stress, which is a measure of the internal resistance of a material to deformity, indicate that bones of gilts from the 2-Ox group were able to withstand higher stresses before fracture. This suggests that bones of gilts supplemented with 2-Ox have a lower susceptibility to deformation.

We also observed positive changes in the metaphyseal trabecular bone morphology after 2-Ox supplementation to growth-retarded gilts. This is in contrast to an earlier study in growth-retarded boars, which demonstrated that the administration of 2-Ox from birth does not improve the morphology of trabecular architecture [20]. On the other hand, our earlier studies have shown that maternal GCs treatment influences trabecular architecture, especially in male piglets [17,19].

Little is known about how dietary 2-Ox, a glutamine precursor, influences the expression of non-collagenous protein. About 10% of non-collagenous proteins are expressed by osteoblasts and chondrocytes, and their expression is considered to be a specific bone turnover marker. Thus, bone formation or remodeling can be assessed through the expression of OC, OPG, RANKL, TIMP-2, VEGF, BMP-2, and GR. Neither non-collagenous proteins expression in the growth plate cartilage and trabeculae nor the alteration in the expression of selected proteins after nutritional modification in growth-retarded gilts has been reported. OC is a typical non-collagenous bone protein reflecting bone formation in GCs therapy [58]. It is considered to be a more sensitive marker of osteoblast and chondrocyte activity in a variety of metabolic connective tissue diseases than serum alkaline phosphatase activity [59]. Additionally, bone and cartilage homeostasis is regulated not only by growth hormone acting through IGF-1, but also by the RANK/RANKL/OPG system [60]. The balance between OPG and RANKL is demonstrated to modulate bone formation and resorption, where OPG is an inhibitor of RANKL and a physiologically negative regulator of osteoclastogenesis [61]. Moreover, BMPs’ signal is important not only for early bone and cartilage development, but also for the maintenance of adult bone homeostasis and reparative processes [62,63]. BMP-2 deficient mice show malformation owing to the disturbance of complex events in the growth plate cartilage, including proliferation, hypertrophic differentiation, and apoptosis [64]. The hypertrophic zone is especially important because the chondrocytes in this region are invaded by blood vessels, osteoblasts, and osteoclasts, in order to initiate the ossification process [65,66]. VEGF is one of the factors, among others, that is released under mechanical load stress and acts via a paracrine or autocrine manner to influence vasculogenesis and angiogenesis. It also stimulates bone mineralization [67]. TIMP-2 plays a key role in the maintenance of the balance between extracellular matrix deposition and degradation in different physiological and pathological processes [52]. Moreover, several studies suggest that osteocytes form the lacunocanalicular system, which allows osteocytes to be exposed to circulating hormones and to modify their microenvironment [68].

The response to the action GCs can be mediated by genes or by non-genomic mechanisms through their receptors [69]. GCs exert their effects directly through glucocorticoid receptors (GRs), which are present on chondrocytes and indirectly through the hypothalamus–pituitary axis [70]. In the present study, more intense immunostaining of the GR was observed in femoral growth plate cartilage in the control growth-retarded gilts, while 2-Ox treatment resulted in weaker GR expression (Figure 8B,b).

Our results showed that long-term postnatal administration of 2-Ox enhanced the expression of OC and OPG in the growth plate cartilage and trabeculae, and reduced the expression of RANKL (Figure 5 and Figure 6). When OPG is over expressed, it can bind RANKL and block RANKL interaction with RANK, suppressing the action of osteoclasts. Moreover, the present findings demonstrated an increase in BMP-2 and TIMP-2 after 2-Ox supplementation in growth-retarded gilts, while a reduction in VEGF expression was observed. Angiogenesis contributes to the repair process, in which new vascular structures are created, and also plays a critical role in the pathogenesis of disease, increasing blood flow by newly formed vessels. Perhaps the current results suggest that 2-Ox may be a modulator of intracellular signaling pathways regulating inflammatory responses, including the induction of angiogenesis and VEGF. This aspect should be further investigated; however, it is consistent with previous results showing a rapid increase in VEGF mRNA levels in response to glutamine deprivation [71].

The dietary supplementation with 2-Ox to growth-retarded gilts showed a protective effect, as evidenced by the morphometry and distribution of proteoglycans (Figure 6). The degradation of proteoglycans can lead to an increase in the risk of degradation, disability, and even pain, as well as in a reduction in quality of life quality, as the physical properties of the articular cartilage (viscosity and elasticity) are determined by the diversity of the components of the matrix. It can influence the distribution of the load through the joint, causing difficulties in movement. Our earlier studies have shown that prenatal overload with GCs may result in predisposition to degradation of articular cartilage [17,19].

## 5. Conclusions

Oral supplementation with 2-oxoglutaric acid to experimentally-induced, intrauterine growth-retarded gilts may increase body weight not only at weaning, but even during subsequent periods of life, when this positive action of 2-Ox is not generally expected. However, the acceleration in body weight gain was not satisfactory from the point of view of the body weight characteristic for a pig in the livestock industry. On the other hand, some improvements in bone metabolism were observed. Dietary supplementation with 2-Ox to growth-retarded gilts showed a regenerative effect on bone mineralization and morphology of trabeculae and articular cartilage. Therefore, the beneficial effects of 2-Ox on health of experimentally induced intrauterine growth retarded gilts are unquestionable. However, specific mechanism of metabolic regulation involving 2-Ox presumably is activated in a state of health problems and differs from those observed in healthy animals. Therefore, despite the fact that 2-Ox is a very promising feed additive, routine dietary supplementation with 2-Ox to pigs in the livestock industry where animals are treated with feed additives dedicated to obtain economic benefits requires further studies.

## Figures and Tables

**Table 1:** Composition of feed mixture used for sows during high pregnancy and lactation.

Ingredients	Content, %
Wheat	19.00
Barley	23.30
Corn	20.00
Soybean meal	17.00
Wheat bran	8.00
Dried alfalfa	6.00
Rapeseed oil	4.00
Calcium carbonate	1.00
Calcium phosphate	0.60
Salt	0.35
Vitamin-mineral premix	0.50
L-Lysine	0.20
DL-Methionine	0.028
L-Threonine	0.03

**Table 2:** Composition of feed mixtures used for offspring.

Ingredients	Content, %	
	Piglets (until 59th Day)	Experimental Gilts (from 60th Day)
Wheat	47.11	30.10
Barley	-	29.00
Corn	15.00	10.00
Soybean meal	18.00	20.00
Wheat bran	-	6.00
Skim milk powder	10.00	-
Dried whey	5.00	-
Rapeseed oil	2.00	2.00
Calcium carbonate	1.10	1.30
Calcium phosphate	0.60	0.50
Salt	0.13	0.28
Vitamin-mineral premix	0.50	0.50
L-lysine	0.30	0.20
DL-methionine	0.14	0.06
L-threonine	0.12	0.06

**Table 3:** Immature, thin collagen content (%) in femora from the control and 2-Ox-administered, nine-month-old, growth-retarded gilts.

Analysed Fragment	Control	2-Ox	*p*-Value
Epiphysieal trabeculae	1.39 ± 0.75	1.45 ± 0.59	0.797
Metaphyseal trabeculae	29.2 ± 10.4	20.2 ± 8.9	0.005
Articular cartilage	1.54 ± 0.83	1.46 ± 1.19	0.721

Data are mean values ± SD. The control group included growth-retarded gilts not supplemented with 2-Ox; the 2-Ox group included growth-retarded gilts supplemented with 2-Ox at a dose of 0.4 g/kg b.w./day.

**Table 4:** Biochemical parameters and the IGF-1 concentration in serum obtained from control and 2-Ox-administered, nine-month-old, growth-retarded gilts.

Parameter	Control	2-Ox	*p*-Value
Cholesterol, mmol/L	1.61 ± 0.13	1.34 ± 0.17	0.012
TG, mmol/L	0.251 ± 0.056	0.255 ± 0.045	0.422
HDL, mmol/L	0.095 ± 0.014	0.101 ± 0.006	0.424
LDL, mmol/L	1.45 ± 0.17	1.11 ± 0.16	0.005
Urea, mmol/L	5.12 ± 0.58	5.64 ± 0.20	0.059
Creatinine, mmol/L	169.4 ± 34.2	173.8 ± 34.3	0.828
Uric acid, mmol/L	0.055 ± 0.013	0.060 ± 0.024	0.691
Albumin, g/L	40.3 ± 3.4	39.0 ± 1.7	0.387
ALT, U/L	33.8 ± 7.4	28.7 ± 9.3	0.313
AST, U/L	52.5 ± 12.7	42.0 ± 14.9	0.218
LDH, U/L	2818.5 ± 447.3	2635.7 ± 638.8	0.578
ALP, U/L	186.8 ± 50.4	223.5 ± 29.6	0.155
Fe, mmol/L	10.7 ± 0.6	11.4 ± 0.7	0.109
Mg, mmol/L	1.11 ± 0.06	1.21 ± 0.11	0.173
P, mmol/L	2.25 ± 0.50	2.33 ± 0.43	0.751
Ca, mmol/L	2.72 ± 0.68	2.80 ± 0.77	0.847
IGF-1, ng/mL	77.8 ± 19.7	92.6 ± 23.8	0.268

Data are mean values ± SD. Control—the control group included growth-retarded gilts not supplemented with 2-Ox; 2-Ox—the 2-Ox group included growth-retarded gilts supplemented with 2-Ox at a dose of 0.4 g/kg b.w./day. TG—triacylglycerol; LDL—low-density lipoprotein; HDL—high-density lipoprotein; ALT—alanine transaminase; AST—aspartate transaminase; LDH—lactate dehydrogenase; ALP—alkaline phosphatase; Fe—iron; Mg—magnesium; P—phosphorus; Ca—calcium; IGF-1—insulin-like growth factor 1.

**Table 5:** Free amino acid concentration in serum obtained from control and 2-Ox-administered, nine-month-old, growth-retarded gilts.

Amino Acid (nmol/mL)	Control	2-Ox	*p*-Value
Arginine	130.3 ± 19.1	123.0 ± 22.4	0.555
Aspartic acid	11.0 ± 0.9	13.7 ± 2.2	0.019
Glutamine	234.7 ± 19.0	313.0 ± 101.5	0.093
Glutamic acid	94.0 ± 9.7	147.2 ± 22.8	0.005
Glycine	1186.7 ± 331.2	736.3 ± 95.6	0.066
Histidine	115.3 ± 17.5	108.5 ± 31.7	0.654
Isoleucine	125.2 ± 23.3	81.3 ± 21.4	0.007
Leucine	265.3 ± 44.4	205.5 ± 30.4	0.021
Lysine	276.3 ± 43.2	297.7 ± 59.6	0.494
Methionine	41.5 ± 7.2	32.2 ± 9.3	0.080
Ornithine	94.8 ± 10.0	116.7 ± 27.3	0.230
Proline	1018.7 ± 94.3	600.0 ± 118.7	<0.001
Serine	165.0 ± 35.8	132.0 ± 39.6	0.066
Threonine	187.2 ± 49.3	124.5 ± 52.0	0.058
Tryptophan	50.0 ± 9.2	52.5 ± 20.7	0.792
Valine	396.3 ± 80.8	299.2 ± 76.5	0.149

Data are mean values ± SD. Control—the control group included growth-retarded gilts not supplemented with 2-Ox; 2-Ox—the 2-Ox group included growth-retarded gilts supplemented with 2-Ox at a dose of 0.4 g/kg b.w./day.
